# Reflection on patients’ experience with orthodontic appliances wear and its impact on oral health related quality of life: observational comparative study

**DOI:** 10.1186/s12903-023-03205-6

**Published:** 2023-07-19

**Authors:** Maram A Abutaleb, Mohammad H Abd El Latief, Mona A Montasser

**Affiliations:** Department of Orthodontics, Faculty of Dentistry, Mansoura University, Mansoura, 35516 Egypt

**Keywords:** Malocclusion, Orthodontic appliances, Oral health, Quality of life

## Abstract

**Background:**

The objective of this study was to explore and compare patient’s experience with the use of a removable functional appliance or fixed orthodontic appliance and its influence on oral health-related quality of life.

**Material and methods:**

This clinical trial included 81 participants having Class II Division 1 and age ranging between 10 and 16 years. The participants were included in any of a three equal groups according to the set inclusion and exclusion criteria; Group 1: patients treated with a Twin-Block functional appliance; Group 2: patients treated with a fixed orthodontic appliance only; and Group 3 (control group): patients not in orthodontic treatment yet. The COHIP SF-19 was used. Patients were given the questionnaire as follows: Group 1: (1) after at least 8 months from starting treatment; (2) after completing phase 1 by 2–3 months without wearing the appliance; Group 2: (1) just before debonding; (2) after finishing the treatment by 2–3 months without any appliances; and Group 3: (1) at the patient’s first visit to the orthodontic clinic; (2) after 2–3 months from the first visit to the orthodontic clinic and before starting any treatment.

**Results:**

The 81 participants were 31 males and 50 females with median age of 13 years. The total COHIP SF-19 scores at baseline were 57 (49–64), 67 (63–72), and 47 (42–53) for the Twin-Block, the fixed appliance, and the malocclusion groups, respectively. Two-month mean scores adjusted to the baseline scores were 64.82 ± 1.15, 65.65 ± 1.47, and 54.45 ± 1.44 for the Twin-Block, the fixed appliance, and the malocclusion groups, respectively.

**Conclusions:**

Both at baseline and two-months (adjusted to the baseline scores), participants in the malocclusion group showed compromised socio-emotional quality of life and reported the poorest total OHRQoL. At the baseline, better socio-emotional and total OHRQoL was reported by the fixed appliance group compared to the Twin-Block group but, after two months both groups gave similar sores. Therefore; patients’ perceptions about their experience with the orthodontic appliance might change.

## Background

Malocclusion is a serious public health problem worldwide. Though malocclusion is neither a life-threatening condition nor a disease, its treatment is highly sought-after [1]. Malocclusion, including Class II Division 1, has a detrimental influence on self-confidence as well as social life, and it has unpleasant psychological implications [2]. On daily function, the effect of a person’s oral health, well-being, and overall quality of life is called oral health-related quality of life [3]. Thus, it has been proposed that malocclusion causes harm to OHRQoL [4, 5].

Some research focuses on patients’ perceptions of the body image in the planning stages of orthodontic treatments [6]. Klages et al. [7] stated that malocclusions have psychological influences on youths and reduce their social interactions and self-confidence. Youths are very concerned about the appearance of their teeth corroborating the theory that dentofacial aesthetics are significant in social interactions as well as psychological well-being [8].

Patients are concerned with orthodontic therapy for improving their appearance, psychological well-being, quality of life, and dental function [9, 10]. The fixed orthodontic appliance is the standard appliance for the treatment of malocclusion. According to Chen et al. [11] in the first month, fixed appliances diminish the quality of life, but function and discomfort levels improve as therapy proceeds. The fixed orthodontic appliance components, such as brackets, may cause pain, an unpleasant appearance, discomfort, and functional restrictions during therapy, which reduce patients’ oral health-related quality of life [10, 12, 13]. However, it is significant to remember the patient’s own perceived expectations of the treatment outcomes in regard to the presenting problem. Patients with their teeth aligned to a high degree who use fixed appliances and who used to be embarrassed by their teeth because of their malocclusion will have a favourable perception [14].

Patients getting removable and fixed orthodontic appliances experienced different levels of pain and discomfort, and eating disturbances; previous research found an inconsistency in the influence of fixed and removable orthodontic treatment techniques on oral health-related quality of life [14–16].

The Twin-Block functional appliance is highly recommended for the first phase treatment of Class II malocclusion in growing patients because of their high acceptance and compliance with the appliance’s wear. The appliance’s two-piece design allows for greater speech and mastication flexibility [17, 18]. In the United Kingdom and the United States, O’Brien et al. state that the Twin-Block appliance is the most used functional appliance [19]. On the other hand, some studies examine the effects of Twin-Block on OHRQoL [14, 20]. Moreover, it is not certain if an individual who uses functional appliances has better oral health results than the one who uses fixed appliances [4]. The comparison between the oral health consequences of functional and fixed orthodontic treatment is a challenge, and additional research is needed before clinical practitioners can choose the kind of orthodontic appliance therapy.

Broder et al. [21] developed the Child Oral Health Impact Profile (COHIP) for measuring OHRQoL in children; there are versions of the COHIP for both children and their carers. The COHIP evaluates the child’s dental health, social and emotional well-being, functional well-being, school environment, self-esteem, and overall OHRQoL. The COHIP was initially designed to assess “oral–facial well-being” in children with age range from 8 to 15 years. The COHIP incorporation of positive aspects of OHRQoL such as confidence and attractiveness is an essential aspect [21]. Later on, Broder et al. [ 22] designed COHIP-SF 19, a short form with 19 questions and three subscales (socio-emotional well-being, functional well-being, and oral health) that retains the original version’s psychometric qualities. The validity and reliability of the COHIP-SF 19 was tested on groups of children including a group of orthodontic patients with age range from 9 to 17 years.

Some short forms are ideal for large surveys since they are easier to use and understand, quicker to complete, and so cost less. Arheiam et al. [22] translated COHIP-SF 19 into Arabic, where his study was conducted for cross-culturally adapting the original English language COHIP-SF 19 to Arabic culture and testing its psychometric properties in a population-based sample of 12-year-old children in schools. The current study examined the experiences of the patients with fixed and removable orthodontic appliances in everyday activities, oral symptoms, and food intake.

## Materials and methods

### Ethical considerations

The Research Ethics Committee of Faculty of Dentistry – Mansoura University provided ethical permission before starting the study (Code: A14030821). The patients’ parents or guardians were thoroughly told about the survey and signed informed consent. The patients’ acceptance to participate in the survey was also obtained.

The following information was gathered for each participant: an allocation number, name, gender, age, telephone number, address, and a unique code. All patients’ information was kept confidential. The data was kept in a secure location, with no one else having access to it except the principal investigator.

### Sample size

It was calculated using Power Analysis and Sample Size Software (PASS, version 15, 2017). NCSS, LLC, Kaysville, Utah, USA, ncss.com/software/pass.

The biostatistician hypothesized an effect size (f = 0.4) between the three research groups based on a review of the literature (no appliance group, fixed appliance group, and clinical appliance group) [23]. A sample size of 27 participants was gathered for each of the three groups whose means would be compared in one-way ANOVA research. Using the F test with a 0.05 significance level, the total sample of 81 patients obtains 89.5% power to detect differences between means over the option of equal means. The effect size (Cohan^,^ s f = m /, which is 0.4), represents the magnitude of the variance in the means.

### Study participants, eligibility criteria, and settings

The sample was recruited with specific inclusion and exclusion criteria. Inclusion criteria: Class II, Division 1 malocclusion, and age ranging between 10 and 16 years; exclusion criteria: patients suffering from craniofacial disorders and patients who did not approve of taking part.

### Study design

A random sample was recruited from the postgraduate orthodontic clinic of the Faculty of Dentistry, Mansoura University.

### Study groups

The study consisted of three groups:

**Group 1 (n = 27)** included patients who used the Twin-Block functional appliances for treatment (Phase 1).

**Group 2 (n = 27)** included patients who were treated with fixed orthodontic appliances only.

**Group 3 (n = 27)** included patients who were not in orthodontic treatment yet. This group was the control group.

### The questionnaire

The main portion of the questionnaire (COHIP-SF 19) comprises 19 items separated into three conceptual subscales: socio-emotional well-being (10 items), functional well-being (4 items), and oral health (5 items). These items are divided into positive and negative answers. Two of the 19 questions utilized positive wording while, 17 of the 19 questions utilized negative wording; after the scoring of the 17 negatively worded items was reversed, the total score ranged from 0 to 76, with the higher score indicating better quality of life. A five-point Likert scale (never = 0, virtually never = 1, occasionally = 2, fairly frequently = 3, and almost always = 4) is used [24].

### Application of the COHIP-SF 19

Participants were given the questionnaire (COHIP-SF-AR) once informed consent was obtained. Though asked by a co-investigator to illustrate any difficult or confusing questions or material that they wanted to understand, all adolescents stated that all of the questions and content were clear. As a result, no further adjustments to the questionnaire’s text were required.

### The questionnaire was administered to the patients at two time points (baseline and after two-month)

**Group 1** (1) Baseline: after at least 8 months from starting treatment; (2) Two-months: after completing phase 1 for 2–3 months without wearing the appliance.

**Group 2** (1) Baseline: just before debonding; (2) Two-months: after finishing the treatment by 2–3 months without any appliance.

**Group 3** (1) Baseline: at the first visit of the patient to the clinic; (2) Two-months: after 2–3 months from the first questionnaire before starting treatment.

### Study interventions

The participants were seated on the dental chair with their parents present. The investigator conducted the participants’ COHIP19-SF-AR questionnaires and asked only the participants to answer.

### Statistical analysis

The data was put into an Excel spreadsheet (v. 2010, Microsoft Corporation, Redmond, WA, USA). IBM-SPSS software (IBM Corp., 2019) was used to enter and evaluate data. IBM SPSS Statistics for Windows, Version 26.0 (IBM Corp., Armonk, NY) N (%) was employed to represent qualitative data. The Chi-square test was employed to determine the relation between two nominal variables. The quantitative data was first checked for normality using the Shapiro-Wilk test, with the data considered normally distributed if p > 0.050. If the data was distributed normally, it was reported as the mean SD (or SE), otherwise as the median (Q1-Q3). For comparing non-normally distributed quantitative data between the three groups, the Kruskal-Wallis H-test was utilised. After adjusting for pre-intervention scores as variables, one-way analysis of covariance (ANCOVA) and its nonparametric counterpart (Quade’s rank ANCOVA) were used to compare post-intervention scores between groups. For analyzing the relationship between a dichotomous variable and a quantitative variable, point biserial correlation was utilized. To determine the direction and intensity of the link between two quantitative variables, Spearman’s correlation was utilized. Cronbach’s alpha was used for measuring internal consistency. If the p value was less than 0.050 for any of the tests conducted, the results were regarded to be statistically important. When necessary, appropriate charts were improved for graphically displaying the findings.

## Results

### Reliability tests for COHIP-SF 19 scores

Reliability tests for the COHIP-SF 19 scores using Cronbach’s alpha for internal consistency of COHIP-19 SF score, showed values mostly ≥ 0.7 for the socio-emotional and total score at Baseline and Two-months, while the values of oral health and functional well-being were < 0.7. Although Cronbach’s alpha values for oral health and functional well-being improved slightly after two months, the values were still below 0.7.

### Age and sex distribution in 3 groups

No statistically significant variation existed in sex distribution across the three groups (p = 0.370). There were 50 females (14, 19, and 17 in groups 1, 2, and 3, respectively). The difference in age between these groups; however, was statistically significant (Kruskal-Wallis H-test, H [2] = 33.764, P = 0.001). Age was statistically significantly greater in group 2 vs. groups 1 and 3 (adjusted P = 0.001), but not between groups 1 and 3 (adjusted P = 1.000). For the whole cohort, the median age was 13 years, ranging from 10 to 16 years (12, 16, and 12 for groups 1, 2, and 3, respectively).

### Baseline COHIP-SF 19 scores in three groups

Baseline COHIP-SF 19 scores in the three groups showed no statistically significant differences in the groups’ oral health and functional well-being scores; however, the socio- emotional well-being score and overall COHIP-SF 19 score were statistically higher in the fixed appliance group followed by the Twin-Block group and finally the malocclusion group, Table 1.

**Table 1:** Baseline COHIP-SF 19 scores in the three groups

Characteristic	Group			Test of significance	
	Group 1	Group 2	Group 3	H [2]	p-value
Oral health score	16 (14–18)	16 (13–18)	15 (14–17)	1.205	0.547
Functional well-being score	14 (12–14)	14 (12–15)	14 (13–16)	4.122	0.127
Socio-emotional well-being score Pairwise comparisons	28 (23–34) A	40 (38–40) B	19 (15–23) C	53.302	**< 0.001**
Total COHIP-SF 19 score Pairwise comparisons	57 (49–64) A	67 (63–72) B	47 (42–53) C	43.988	**< 0.001**

Notes: Data is median (Q1-Q3), test of significance is Kruskal-Wallis H-test. Pairwise comparisons are presented as letters (similar letters = insignificant difference, different letters = significant difference).

### Two-months COHIP-SF 19 scores in the three groups

Two-months COHIP SF-19 scores in the three groups showed that overall, scores of each of the four subscales were significantly lower in the malocclusion group than the Twin-Block and fixed appliance groups (except for oral health scores and functional well-being scores, where there was no significant difference between the fixed appliance and malocclusion groups, Table 2.

**Table 2:** Two-months COHIP-SF 19 scores in the three groups

Characteristic	Group			Test of significance	
	Group 1	Group 2	Group 3	H [2]	p-value
Oral health score	18 (16–20) A	16 (14–18) A, B	16 (14–17) B	6.399	**0.041**
Functional well-being score	16 (14–16) A	16 (14–16) A, B	14 (14–16) B	6.421	**0.040**
Socio-emotional well-being score (Pairwise comparisons)	34 (28–38) A	40 (40–40) B	20 (17–24) C	56.914	**< 0.001**
Total COHIP-SF 19 score (Pairwise comparisons)	66 (57–72) A	72 (68–73) A	49 (46–55) B	45.621	**< 0.001**

Notes: Data is median (Q1-Q3), test of significance is Kruskal-Wallis H-test. Pairwise comparisons are presented as letters (similar letters = insignificant difference, different letters = significant difference)

### Correlations between baseline COHIP SF-19 scores, age, and sex

Correlation results showed a statistically significant positive correlation between oral health scores and sex as female gave higher scores than males at baseline, Table 3a. Also, there was a statistically significant positive correlation between total oral health scores and age in the malocclusion group after two months from the first questionnaire, Table 3b.

**Table 3a:** Correlations between baseline COHIP SF-19 scores, age, and sex

Characteristic	OHS		FWS		SEWS		Total scores	
	r	p	r	p	r	p	R	P
**Group 1 (Twin-Block, n = 27)**								
Age (years)	-0.116	0.563	-0.023	0.910	0.072	0.721	0.042	0.835
Sex	0.165	0.411	-0.278	0.161	0.178	0.375	0.130	0.520
**Group 2 (Fixed appliance, n = 27)**								
Age (years)	-0.116	0.563	-0.023	0.910	0.072	0.721	0.042	0.835
Sex	0.419	0.030	-0.043	0.832	-0.209	0.295	0.028	0.889
**Group 3 (Control group, n = 27)**								
Age (years)	-0.288	0.146	-0.093	0.643	-0.126	0.536	-0.205	0.306
Sex	0.050	0.804	0.172	0.392	-0.252	0.204	-0.163	0.416

**Table 3b:** Correlations between post-intervention COHIP SF-19 scores, age, and sex

Characteristic	OHS		FWS		SEWS		Total scores	
	r	P	r	P	r	P	R	P
**Group 1 (Twin-Block, n = 27)**								
Age (years)	-0.076	0.706	-0.092	0.648	-0.155	0.439	-0.147	0.466
Sex	0.185	0.357	-0.285	0.150	0.108	0.593	0.108	0.593
**Group 2 (Fixed appliance, n = 27)**								
Age (years)	-0.076	0.706	-0.092	0.648	-0.155	0.439	-0.146	0.466
Sex	-0.058	0.772	0.099	0.624	-0.211	0.292	-0.088	0.662
**Group 3 (Control group, n = 27)**								
Age (years)	-0.401	0.038	0.105	0.601	-0.186	0.353	-0.273	0.169
Sex	-0.077	0.701	-0.183	0.362	-0.119	0.553	-0.178	0.375

Notes: r = correlation coefficient (Point biserial for sex, and Spearman’s for others). OHS = oral health score. FWS = functional well-being score. SEWS = socio-emotional well-being score. Total = Total COHIP SF-19 scores

### Comparisons between two-months scores of the three groups adjusted to the baseline scores

Higher oral health scores were seen in the Twin-Block group than in the fixed appliance group followed by the malocclusion group; nonetheless, this difference, statistically, was not important (Table 4). It also showed a higher functional well-being score in the Twin-Block group than in both the fixed appliance and malocclusion groups. This difference in Two-months functional well-being scores after controlling (adjusting) for Baseline scores was statistically significant. Pairwise comparisons revealed that the difference was statistically significant for Twin-Block and malocclusion groups (p-value = 0.005 [Quade’s rank ANCOVA], and 0.013 [one-way ANCOVA]). Results also showed higher socio-emotional well-being scores in the fixed appliance group than in both the Twin-Block and malocclusion groups. The difference was significant statistically between the fixed appliance group and the malocclusion group (p-value = 0.015 [Quade’s rank ANCOVA], and < 0.001 [one-way ANCOVA]). Finally, results also showed the highest total COHIP SF-19 scores in the fixed appliance group, followed by the Twin-Block group and then the malocclusion group. Pairwise comparisons revealed that the difference was statistically significant between both the Twin-Block and the fixed appliance groups and the malocclusion group (p-value = 0.001 [Quade’s rank ANCOVA], and < 0.001 [One-way ANCOVA]) and (p-value = 0.025 [Quade’s rank ANCOVA], and < 0.001 [One-way ANCOVA]) respectively, Table 4.

**Table 4:** Two-months scores adjusted to the baseline scores in the three groups

**Group**	**Means and variability**				**One-way ANCOVA**		**Quade’s rank ANCOVA**	
	**Unadjusted**		**Adjusted**		**F**	**P-value**	**F**	**p-value**
	**Mean**	**SD**	**Mean**	**SE**				
**Oral Health**								
Group 1	17.37	2.79	17.33	0.458	2.921	0.060	3.044	0.053
Group 2	16.19	2.15	16.15	0.458				
Group 3	15.78	2.24	15.85	0.459				
**Functional well-being**								
Group 1	15.26	1.23	15.37	0.269	4.462	**0.015**	5.366	**0.007**
Group 2	14.96	1.43	14.96	0.265				
Group 3	14.33	1.57	14.23	0.296				
**Socio-emotional well-being**								
Group 1	31.96	7.38	32.04	0.928	13.383	**< 0.001**	4.336	**0.016**
Group 2	39.11	2.01	34.73	1.314				
Group 3	19.96	5.48	24.26	1.303				
**Total OHRQoL**								
Group 1	64.59	8.91	64.82	1.149	17.018	**< 0.001**	7.729	**0.001**
Group 2	70.26	4.61	65.65	1.471				
Group 3	50.07	6.26	54.45	1.443				

Notes: ANCOVA = analysis of covariance. SD = standard deviation. SE = standard error

## Discussion

In the current study, the children took the same questionnaire a second time after 2–3 months to examine the effect of time on their perceptions. The time interval was selected to be 2–3 months since this was a period regarded as not long enough for the participant’s OHRQoL to alter considerably but long enough for the participants to forget their earlier replies.

The COHIP-SF 19 form that was used in this study was validated and proven to be reliable in both its original English version [24] as well as in its Arabic version [22]. Thus, COHIP-SF 19 is a psychometrically sound tool for measuring oral health-related quality of life in preadolescents and adolescents. In the current study, when the internal consistency of the COHIP-19 SF scores was assessed, using Cronbach’s alpha, it was observed that the socio-emotional and total scores were satisfactory; however, the functional well-being subscales and oral health displayed unsatisfactory results. Results were similar at baseline and after two months. This is in agreement with other published data found on both the Arabic [22] and Japanese versions of COHIP-SF 19 [25]. A low alpha value might be due to the small number of question [26].

The findings of the current study presented no statistically significant differences in sex distribution among the three groups. This was advantageous because the results about the effect of sex on the subjects’ perceptions were mixed in previous studies. Thiruvenkadam et al. [26] evaluated the oral health-associated quality of life of children who sought orthodontic treatment, and found no statistically significant difference in COHIP SF scores between girls and boys and The results of the current study agrees as the only positive correlation was observed between oral health scores and sex in the fixed appliance group at base line where the scores were higher in females than in males. In contrast, the COHIP-SF-19 levels were lower in girls than in boys in the New Caledonia COHIP-SF-19 validation study while, the Japanese COHIP-SF-19 validation research indicated greater results for girls [25, 27]. Previous research that used the Chinese COHIP-SF 19 noticed no gender differences in total or socio-emotional well-being subscores; however, females had higher functional well-being and oral health subscores than males [28].

General health-related quality of life (HRQoL) has often been reported to be higher in children than adolescents [29, 30]. However; in the Japanese children, the increased functional well-being subscores in the older group while the social-emotional well-being subscores were lower in the older age group. It is worth mentioning that COHIP-SF 19 was suggested to be used for comparing OHRQoL in orthodontic patients, with age range from 9 to 17 [24]. The results of the current study showed almost no significant correlation between age and OHRQoL subscales within each of the three groups which is most probably related to the homogenously selected groups.

Just after finishing the treatment (baseline assessment), the results of the present study indicated an important difference between the three groups, with the malocclusion group expressing the worst experience, followed by the Twin-Block group and then the fixed appliance group. At Two-months, compared to the fixed appliance group and the Twin-Block group, the malocclusion group had the worst quality of life, but the fixed appliance and the Twin-Block groups had similar quality of life although the scores were slightly higher in the fixed appliance group. Previous studies documented the psychological effect of malocclusion on patients’ quality of life [31, 32]. Similarly, in an assessment for OHRQoL of orthodontic patients using fixed and Twin-Block appliances done by Alzoubi et al. [14] patients reported that their quality of life was improved; after treatment, there were no statistically noticeable differences between both groups, which suggests that the perceived impact of removable functional appliances on OHRQoL may be overestimated by clinicians. Treating the malocclusion enhances the quality of life regardless of the appliance. The findings of the current study showed that after two months, patients’ reflections on their treatment experience were not different between the fixed and removable appliances. As time passes, patients’ perceptions change.

Just after finishing the treatment (the baseline assessment), patients in all groups had similar functional well-being, a situation that changed after two months, with the best functional well-being in the Twin-Block group followed by the fixed appliance group and then the malocclusion group. Looking at the socio-emotional well-being differences, they were obvious. The malocclusion group had the lowest scores both at the baseline and after two months in comparison to the other two groups. Just after finishing the treatment, patients in the fixed appliance group showed significantly higher scores than the Twin-Block group, which could be attributed to the nature of the appliance; removable vs. fixed appliance. Patients in the fixed appliance group might have been happier to realize that they are in the last stage of treatment and about to remove the appliance finally. After two months without the appliance, both the fixed appliance and functional appliance groups had similar socio-emotional well-being.

In the present study, the findings of the malocclusion group were consistent with those of previous studies that showed that a great number of patients who required orthodontic treatments felt ashamed and inferior; the more the need for treatment, the greater the person’s humiliation [33]. De Oliveira and Sheiham [34]conducted a study on Brazilian adolescents and revealed that adolescents who had malocclusion and were treated, had a better oral OHRQoL than those who were still under treatment or who never had treatment. Furthermore, Helm et al. [35] stated that orthodontic patients (not only as children but also as adults) feel shame and suffer from self-consciousness. Another study revealed that young people with greater treatment requirements were more socially isolated than those with lower treatment needs [36]. Also, previous studies found that orthodontic patients are more concerned with aesthetic and social issues than with interference with daily tasks [37]. However, some studies have shown no relationship between malocclusion and self-consciousness or shame [38].

One of the limitations of the study is the inability to adhere to one gender or design different sex groups that would have been more insightful; however, the results showed no statistical difference in sex distribution among the groups. The same applies to age; it would have been better to have groups with no statistical age range differences; however, COHIP-SF 19 is suitable for a wide age range, as elaborated upon in the discussion section.

## Conclusions

Both at baseline and Two-months (adjusted to the baseline scores), participants in the malocclusion group showed compromised socio-emotional quality of life and reported the poorest total OHRQoL. At the baseline, better socio-emotional and total OHRQoL was reported by the fixed appliance group compared to the Twin-Block group but, after two months both groups gave similar sores. Therefore; patients’ perceptions about their experience with the orthodontic appliance might change. Just after finishing the treatment, patients in all groups had similar functional well-being, while after two months; the best functional well-being was in the Twin-Block group followed by the fixed appliance group, and then the malocclusion group.

## Data Availability

The datasets used and/or analyzed during the current study are available from the corresponding author upon reasonable request.

## Abbreviations

COHIP SF-19: Child Oral Health Impact Profile Short Form − 19
COHIP-SF-AR: Child Oral Health Impact Profile -Short Form-Arabic
COHIP: Child Oral Health Impact Profile
OHRQoL: Oral Health Related Quality of Life
